# Anatomic study of the medial side of the ankle base on the joint capsule: an alternative description of the deltoid and spring ligament

**DOI:** 10.1186/s40634-019-0171-y

**Published:** 2019-01-28

**Authors:** Kentaro Amaha, Akimoto Nimura, Reiko Yamaguchi, Natnicha Kampan, Atsushi Tasaki, Kumiko Yamaguchi, Ryuichi Kato, Keiichi Akita

**Affiliations:** 1 Department of Clinical Anatomy, Graduate School of Medical and Dental Sciences, Tokyo Medical and Dental University, Tokyo, Japan; 2 Department of Orthopaedic Surgery, St. Luke’s International Medical center, Tokyo, Japan; 3 Department of Functional Joint Anatomy, Graduate School of Medical and Dental Sciences, Tokyo Medical and Dental University, 1-5-45 Yushima, Bunkyo-ku, Tokyo, 113-8519 Japan; 4 JA Kyosai Research Institute, Tokyo, Japan

**Keywords:** Anatomy, Medial ankle, Deltoid ligament, Spring ligament, Capsule

## Abstract

**Background:**

Adult acquired flatfoot deformity (AAFD) is caused by impaired medial ligamentous structures and posterior tibialis tendon dysfunction (PTTD). Although degeneration and trauma could separately cause AAFD, how these factors interact in the pathomechanism of AAFD is unclear. The joint capsule in the medial ankle is considered an important structure, providing passive stability by limiting joint movement. Previous reports on the joint capsule suggest its involvement in pathological changes of the ankle, but because of the high priority placed on the ligaments, few reports address the ankle joint from the joint capsule standpoint. The current study aimed to anatomically examine the medial ankle joint, focusing on the deltoid and spring ligaments in perspective of the joint capsule.

**Methods:**

We conducted a descriptive anatomical study of 19 embalmed cadavers (mean 82.7 years, range 58 to 99). We included 22 embalmed cadaveric ankles. We detached the joint capsule in 16 ankles from the anterior to posteromedial joint, analyzed the capsular attachments of the ankle and adjacent joints, and measured the widths of the bony attachments. We histologically analyzed the joint capsule using Masson’s trichrome staining in 6 ankles.

**Results:**

The capsule could be separated as a continuous sheet, including 3 different tissues. The anterior capsule was composed of fatty tissue. Between the medial malleolus and talus, the capsule was strongly connected and was composed of fibrous tissue, normally referred to as the deep deltoid ligament. The tibial attachment formed a steric groove, and the talar side of the attachment formed an elliptical depressed area. On the medial part of the subtalar and talonavicular joints, the capsule covered the joints as cartilaginous tissue, normally referred to as the superomedial ligament of the spring ligament. The outer side of the cartilaginous and fibrous tissue formed the sheath floor of the posterior tibialis tendon. Histological analysis revealed three different tissue types.

**Conclusions:**

The capsules of the ankle, subtalar, and talonavicular joints could be detached as a continuous sheet. The deltoid and the superomedial ligament of the spring ligaments could be interpreted as a part of the continuous capsule, which had different histological features.

**Level of evidence:**

Descriptive Laboratory Study.

## Background

The etiology of adult acquired flatfoot deformity (AAFD) has been considered to be failure of the medial arch stabilizer, primarily posterior tibialis tendon dysfunction (PTTD) (Funk et al. 1986; Kohls-Gatzoulis et al. 2004). A recent study showed that failure of the ligamentous structures, including the spring and deltoid ligaments, is also involved in AAFD, along with PTTD (Deland et al. 2005; Orr and Nunley 2nd 2013; Tryfonidis et al. 2008). To elucidate the pathologic change underlying the initiation of AAFD, the anatomical association between the dynamic stabilizers of the medial ankle, such as the posterior tibialis tendon, and the static ones, such as the spring and deltoid ligaments, should be understood.

Previous reports have described the deltoid and spring ligaments to consist of variable, complex patterns (Boss and Hintermann 2002; Campbell et al. 2014; Panchani et al. 2014; Vadell and Peratta 2012). Regarding the deltoid ligament, the morphology of its components is confusing because of the difficulty of differentiation from adjacent tissues. Additionally, its morphological association with the spring ligament remains unknown. Because of the high priority placed on the ligaments, there are very few reports addressing the ankle joint from the standpoint of the joint capsule, which should theoretically exist at the deep layer of the medial ankle joint, as is seen in other joints.

Generally, the structures surrounding a synovial joint comprise a synovial joint capsule and a fibrous joint capsule (Fick 1904). Similarly, the joint capsule in the medial ankle has been considered an important structure, providing passive stability by limiting joint movement (Ralphs and Benjamin 1994). Previous reports on the joint capsule of the shoulder, elbow, and knee joints have provided information on the etiologies of some pathologies and have helped better understand the stabilizing mechanism of these joints (Nasu et al. 2017; Nimura et al. 2014; Nimura et al. 2012). We hypothesized that the deltoid and spring ligaments could be alternatively described in perspective of the joint capsule regarding the composition of the deep layer of the medial ankle joint. The aim of the current study is to anatomically examine the medial ankle joint, focusing on the deltoid and spring ligaments in perspective of the joint capsule.

## Methods

This is a descriptive anatomic study using embalmed cadavers. We included 27 ankles from 24 cadavers. We excluded 1 specimen that appeared to have dry gangrene of the toes, and 4 specimens with severe deformity and remarkable osteoarthritis seen after dissecting the joints. There were no specimens with significant scars of surgical treatment on the medial ankle. We ultimately evaluated 22 ankles (13 right, 9 left) from 19 cadavers (7 male, 12 female; mean age, 82.7 years, range 58–99). Of these, we analyzed 16 ankles macroscopically and the remaining 6, histologically. All cadavers were fixed in 8% formalin and preserved in 30% ethanol. The cadavers were donated to the Department of Anatomy of the School of Medicine, Tokyo Medical and Dental University. The entire foot and ankle with soft tissues were obtained by sectioning the distal third of the lower limb.

First, to expose the joint capsule and the tendons of medial side of the ankle joint, we removed the skin, subcutaneous tissues, anterior extensor tendons, and retinaculum. We observed the joint capsule carefully after removing the neurovascular bundle, the posterior tibialis (PT) tendon, the flexor digitorum longus (FDL) tendon, and the flexor hallucis tendon. Next, we detached the joint capsule from the center line of the anterior ankle joint along the long axis of the tibia posteromedially, analyzed the capsular attachments on the ankle and adjacent joints, and measured the widths of the bony attachments of the joint capsule. Each measurement was performed twice, and an intra-class correlation coefficient (ICC) was calculated to test reliability of the measurements. ICCs were determined by evaluating the measurement process (EMP) analysis using JMP 12.0.1 (SAS Institute Inc., Cary, NC, USA).

In addition, we used 6 ankles for histological analyses. We removed the skin and subcutaneous tissues. We made blocks including the distal tibia, medial side of the talus, and proximal side of the navicular using the diamond band pathology saw (EXAKT 312; EXAKT Advance Technologies, Norderstedt, Germany). We decalcified the tissues for 1 week in a solution containing aluminum chloride, hydrochloric acid, and formic acid, as described by Plank and Rychlo (Plank and Rychlo 1952). After decalcification, we sectioned the blocks to divide them into several parts parallel to the PT tendon. We dehydrated the sections with a graded ethanol series. After dehydration, we embedded them in paraffin and serially sectioned them (5-μm thickness) parallel to the sectioned plane. We stained the sections with Masson trichrome.

## Results

### Macroscopic observation of the joint capsule and its attachment in the medial ankle joint

**Fig. 1:**
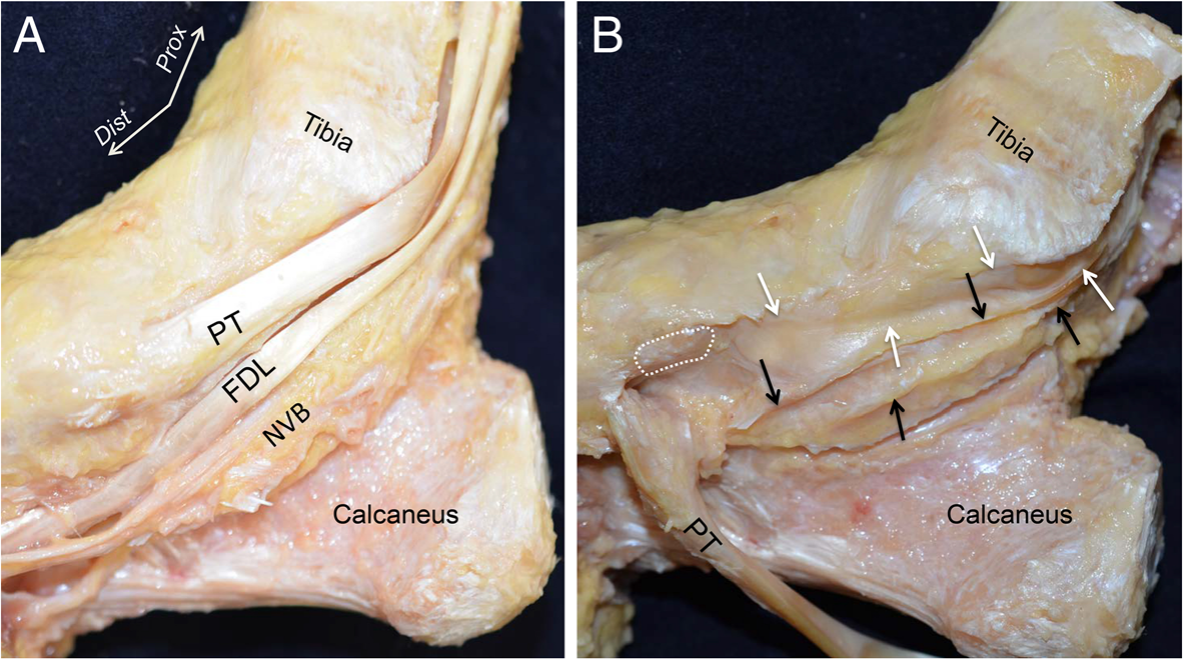
Outer appearance of the medial joint capsule. **a** The medial side of the right ankle joint. The courses of the posterior tibialis (PT) and the flexor digitorum longus (FDL) tendons and the neurovascular bundle (NVB) are shown after removing the subcutaneous tissue and the superficial sheath of the tendons. **b** To expose the outer appearance of the medial joint capsule, the PT and FDL tendons and the NVB were removed. The navicular insertion of the PT (*white dotted area*) was detached and reflected in the plantar direction. The sheath floors of the PT (*white arrows*) and FDL (*black arrows*) tendons are continuously connected with the anterior part of the joint capsule. *Dist*, distal; *Prox*, proximal

The PT tendon ran around the posterior edge of the medial malleolus and inserted into the navicular. Posterior and parallel to the PT tendon, the FDL tendon and the neurovascular bundle ran on the posteromedial side of the ankle joint (Fig. 1a). To expose the outer appearance of the joint capsule of the medial ankle, we removed the tendons and the neurovascular bundle. A portion of the PT tendon inserted into the navicular, and another portion travelled along the bottom side of the navicular. The medial capsule formed the sheath floor under the PT tendon and FDL tendon (Fig. 1b).

**Fig. 2:**
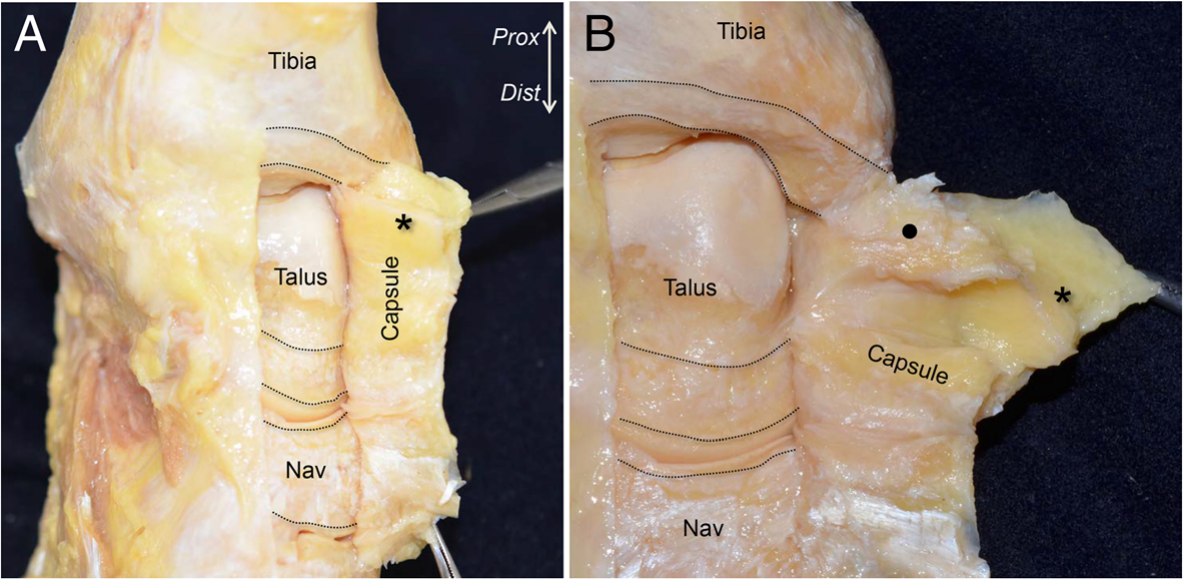
Attachments and inner appearance of the anterior capsule of the anteromedial ankle. **a** To expose the attachment of the joint capsule, we detached it from lateral to posteromedial. The superior aspect of the ankle joint is shown. The *black dotted lines* indicate the dimension of the capsular attachments. The anterior part of the joint capsule consisted of fatty tissue (asterisk). **b** The capsule was detached medially. The anterior fatty tissue of the capsule changed to fibrous tissue (black circle) at the anteromedial aspect of the ankle joint. *Prox,* proximal; *Dist,* distal; *Nav*, navicular

To observe the attachments of the joint capsule on the anterodistal tibia, the dorsum of the talus and navicular, we detached the joint capsule from anterior to posteromedial. On the tibia, the joint capsule attached to the distal edge of the tibia with constant width. On the talus, the joint capsule attached proximal to the talonavicular joint. On the navicular, the joint capsule occupied most of the bony surface between the talonavicular and naviculocuneiform joints. The anterior part of the joint capsule formed a continuous membrane, which predominantly consisted of fatty tissues, and overlaid both the ankle and talonavicular joints (asterisk in Fig. 2a). The capsule was detached in the posteromedial direction. At the anterior colliculus of the medial malleolus, the fatty capsule changed to be a fibrous structure (black circle in Fig. 2b) and strongly attached to the tibia.

**Fig. 3:**
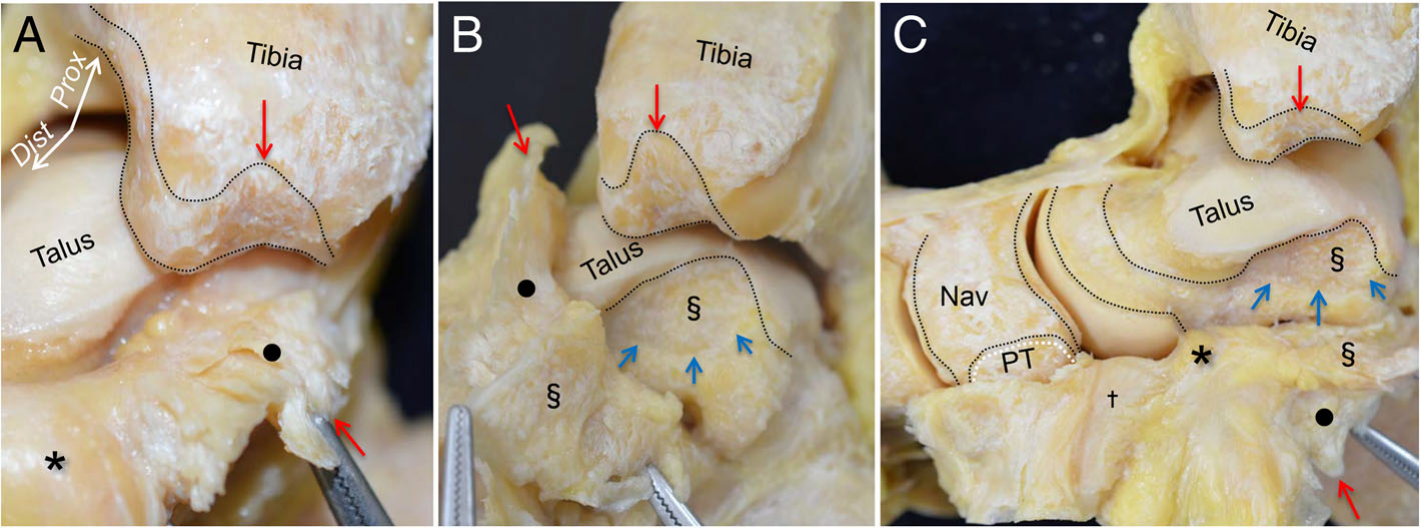
Detailed observation of the joint capsule in the medial ankle. **a** The medial part of the capsule appeared fibrous (*black circle*). There is a clear qualitative difference between the anterior fatty tissue (*asterisk*) and the medial fibrous tissue. Red arrows indicate the corresponding bone and detached capsule. **b** The fibrous capsule occupied the space of the intercolliculus groove like a mortise joint. The corresponding side of the fibrous capsule (section mark in Fig. 3b) widely attached to the depressed area of the medial side of the talus (blue arrows), which formed an ellipse. **c** The posteromedial fibrous capsule extended distally, and changed to cartilaginous tissue (*dagger*). The cartilaginous tissue covered the talus head, and attached to the proximal portion of the PT tendon insertion (*white dotted line*). Similar marks represent the attachments of the capsule and corresponding parts of the capsule. *Prox,* proximal; *Dist,* distal; *Nav*, navicular; *PT*, insertion of the posterior tibialis tendon

Next, the capsule was detached in the posterior and distal direction. The fibrous capsule of the medial ankle was firmly attached at the top of the intercolliculus groove (red arrow in Fig. 3a). The corresponding side of the fibrous capsule (section mark in Fig. 3b) was widely attached to the depressed area of the medial side of the talus (blue arrows), which formed an ellipse shape (Fig. 3b). Additionally, the joint capsule was detached distally as a continuous membrane. At the medial part of the talonavicular joint, the property of the capsule changed from fibrous to cartilaginous (dagger in Fig. 3c) and was interspersed with fatty tissue coming from the anterior capsule (asterisk in Fig. 3c). The cartilaginous capsule covered the talus head, and attached proximal to the navicular insertion of the PT tendon (white line).

### Entire appearance of the joint capsule of the medial ankle

**Fig. 4:**
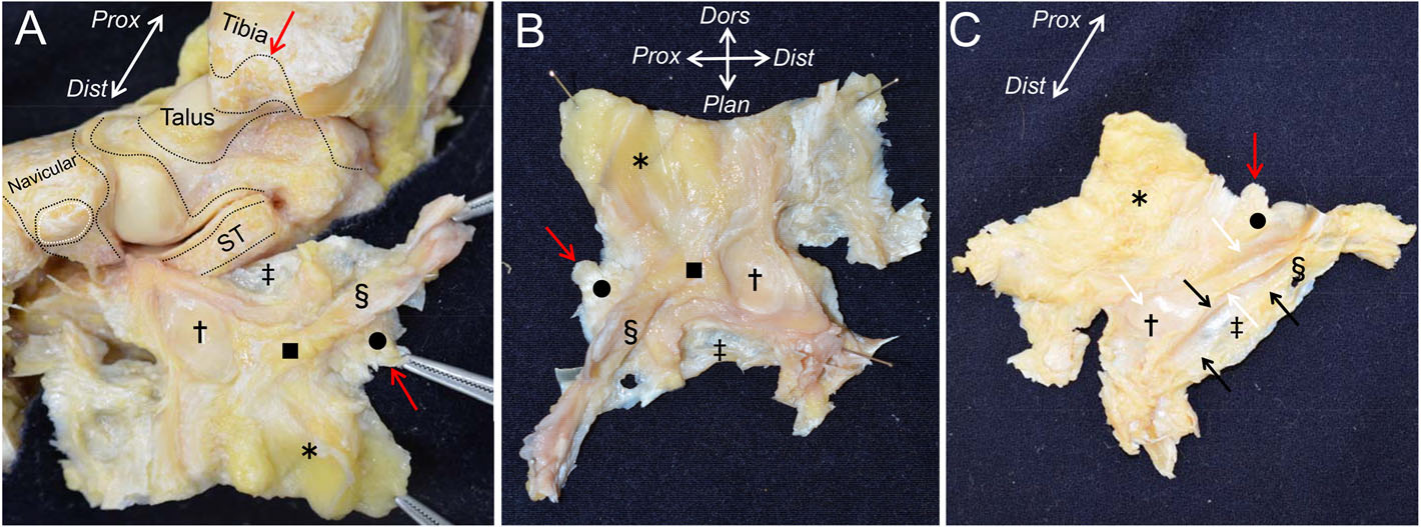
Whole appearance of the joint capsule. **a** The whole flipped continuous capsule remaining attachment to the calcaneus is shown. The black dotted area indicates the attachment of the joint capsule. The white dotted area indicates the insertion of the posterior tibialis (PT) tendon. Red arrows indicate the corresponding bone and detached capsule. **b** The inner appearance of the continuous capsule is shown after being completely detached from the calcaneus. The continuous capsule consists of 3 different tissues: fatty tissue (*asterisk*), fibrous tissue (*black circle*), and cartilaginous capsule (*dagger*). The fatty tissue extended medially and attached between the fibrous and the cartilaginous tissue (*black square*). **c** The outer appearance of the continuous capsule. The part of the fibrous tissue (*black circle*) and the cartilaginous tissue (*dagger*) formed the sheath floor of the PT tendon (*white arrows*), and the part of the fibrous tissue (*section mark*) formed the sheath floor of the flexor digitorum longus tendon (*black arrows*). *Prox,* proximal; *Dist,* distal; *Plan*, plantar; *Dors*, dorsal; *PT*, insertion of the posterior tibialis tendon; *Nav*, navicular; *ST*, sustentaculum tali; double dagger, capsule corresponding to sustentaculum tali

To comprehensively observe the entire capsule of the ankle, subtalar, and talonavicular joints, we completely detached them from the sustentaculum tali of the calcaneus (double dagger in Fig. 4a) and expanded them as a flat continuous sheet (Fig. 4b). The inner appearance of the continuous capsule showed 3 different properties. First, the anterior part of the continuous capsule, corresponding to the anterior ankle and dorsal talonavicular joints, appeared to be fatty tissue (asterisk). Second, the posteromedial part of the continuous capsule, corresponding to the medial ankle joint, appeared to be fibrous and steric tissue (black circle in Fig. 4). Third, the distal part of the continuous capsule, corresponding to the medial side of the talus head and the talonavicular joint, appeared to be cartilaginous tissue (dagger in Fig. 4). The anterior fatty tissue extended to the medial side and attached between the fibrous and the cartilaginous tissues (black square in Fig. 4a, b). As this fatty tissue lay beneath the sheath floor of the PT tendon (white arrows in Fig. 4c), we could not observe its outward appearance (Fig. 4c). The outer appearance of the continuous capsule, the sheath floor of the PT tendon (white arrows in Fig. 4c), and the FDL tendon (black arrows in Fig. 4c) consisted of the outside of the fibrous and steric tissue, and the cartilaginous tissue. The capsular attachment of the sustentaculum tali was directly beneath the sheath floor of the FDL tendon. These anatomical findings were consistently preserved in all specimens, even in the relatively younger ones (58 and 60 years old).

**Fig. 5:**
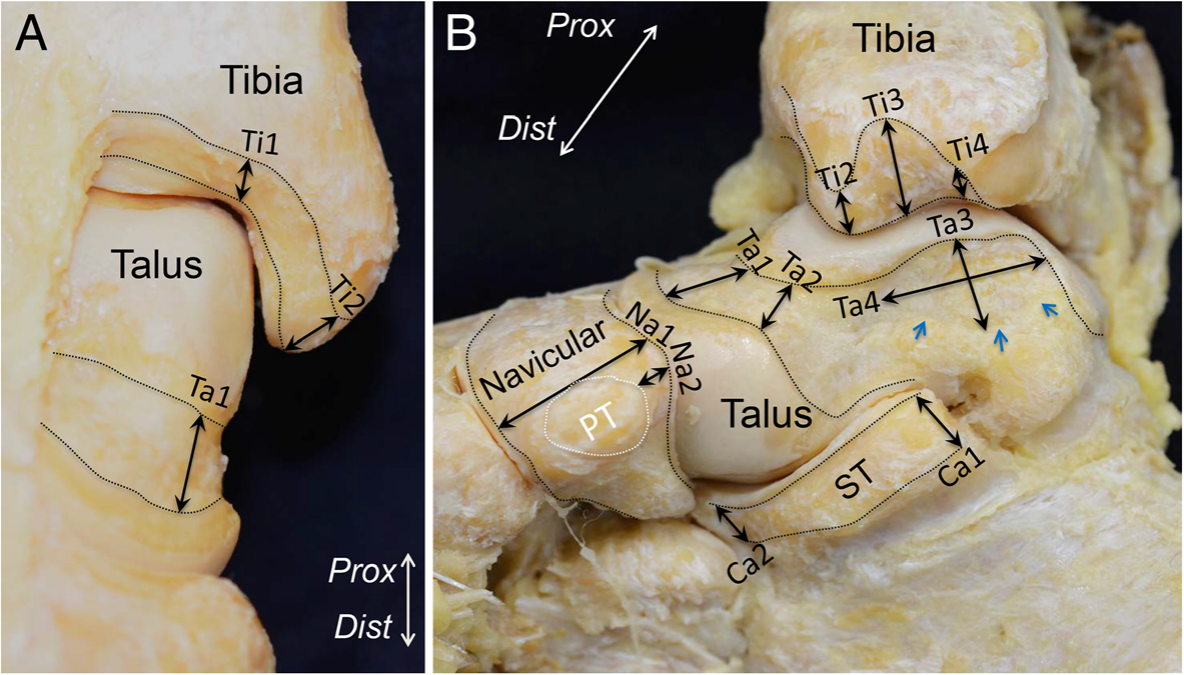
Measurements of the width of the capsule attachment. Detailed data are shown in Table 1. **a** Attachment of the anterior part of the continuous capsule. **b** Attachment of the medial part of the continuous capsule. The black dotted line indicates the attachment of the joint capsule. The white dotted line indicates insertion of the posterior tibialis tendon. *Prox,* proximal; *Dist,* distal; *PT*, insertion of the posterior tibialis tendon; *ST*, sustentaculum tali

The measured widths of the attachments of the continuous capsule are shown in Fig. 5. In the tibia, the capsular attachment (13.8 ± 1.2 mm, average ± standard deviation, Ti3 in Fig. 5) was largest at the intercolliculus groove, which corresponded to the fibrous steric structure in the continuous capsule. On the talus, the dimension of the depressed area that corresponded to the fibrous and steric structures was 8.6 ± 1.6 mm along the short axis and 17.9 ± 3.0 mm along the long axis (Ta3 and Ta4 in Fig. 5b). All measured data are reported in Table 1. ICCs were calculated to compare the reliability of measurements, which ranged from 0.82 to 0.96. All scores were above 0.80, which is considered excellent agreement.

**Table 1:** Measurements of the width of the capsular complex

Location of the measurements^a^	Average and standard deviation (mm)
Capsule attachment on the tibia	
Medial edge of talar plafond (Ti1)	5.0 ± 1.7
Anterior colliculus (Ti2)	10.3 ± 1.7
Intercolliculus (Ti3)	13.7 ± 1.3
Posterior colliculus (Ti4)	2.9 ± 0.9
Capsule attachment on the talus	
Extension of the medial edge of talar plafond (Ta1)	8.1 ± 1.6
Posteromedial corner of anterior talonavicular joint cartilage (Ta2)	7.2 ± 2.3
Short axis (Ta3)	8.9 ± 1.4
Long axis (Ta4)	17.5 ± 3.2
Capsule attachment on the navicular	
Lateral edge of insertion of PT tendon (Na1)	19 ± 2
Proximal part of insertion (Na2)	5.1 ± 2.1
Capsule attachment on the calcaneus	
Posterior edge of sustentaculum tali (Ca1)	8.5 ± 1.4
Anterior edge of sustentaculum tali (Ca2)	7.1 ± 1.6

^a^Locations of the measurements are depicted in Fig. 5

### Histological analysis of the joint continuous capsule

**Fig. 6:**
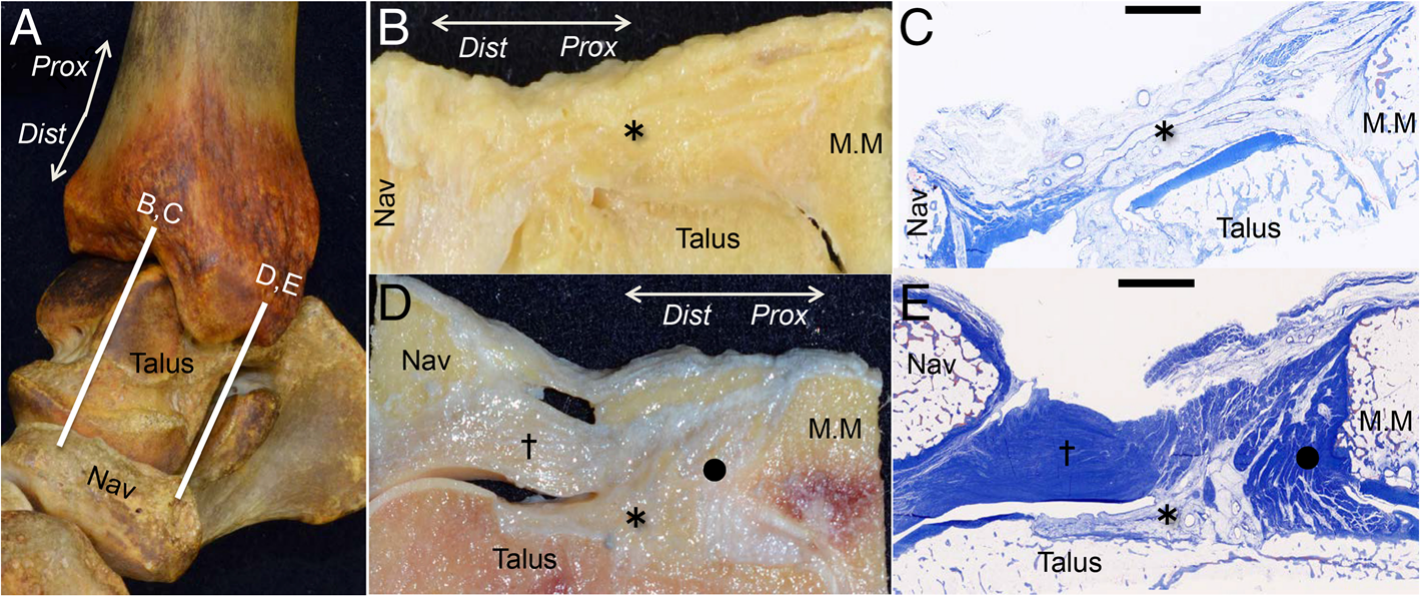
Histological analysis of the joint continuous capsule. **a** The bone model of the right medial ankle shows the locations of the anterior section (**b**, **c**) and anteromedial section (**d**, **e**). **b** and **d** show the macroscopic views of (**c**) and (**e**), which were stained with Masson’s trichrome, respectively. **b**, **c** At the anterior part of the ankle, the fatty tissue of the continuous capsule (asterisk) covered the ankle and talonavicular joints. **d**, **e** At the anteromedial part of the medial ankle, the fibrous tissue (black circle) connected between the medial malleolus and the medial talus. The fatty tissue (asterisk) occupied the space between the fibrous and the cartilaginous tissues. The cartilaginous tissue (dagger) covered the talus and connected the medial malleolus and the medial navicular. *Prox,* proximal; *Dist,* distal; *Nav*, navicular; *MM*, medial malleolus; scale bar, 5 mm

To histologically analyze the joint continuous capsule, sagittal sections were made at the anterior and anteromedial portions (Fig. 6a). At the anterior part of the ankle, the fatty tissue (asterisk in Fig. 6b, c) covered the ankle and talonavicular joints. At the anteromedial part, the fibrous tissue (circle in Fig. 6d, e) connected between the medial malleolus and the medial talus as a collagenous structure. The cartilaginous tissue (dagger in Fig. 6d, e) covered the talus, and connected between the medial malleolus and the navicular, which was superficial to the fibrous tissue (dagger) and fatty tissue (asterisk).

## Discussion

**Fig. 7:**
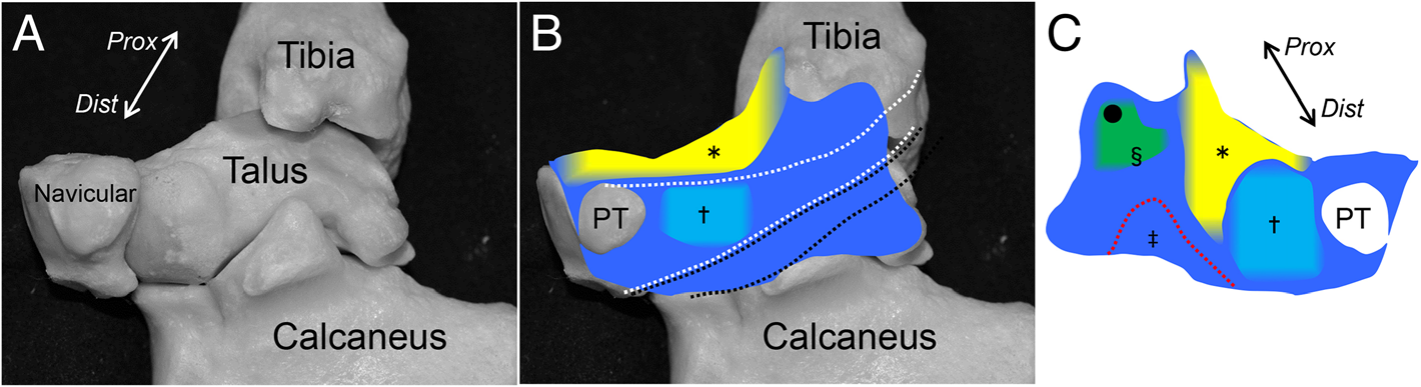
Schematic illustrations of the continuous capsule on the medial ankle. **a** Underlying bones of the right medial ankle on the continuous capsule. **b** The outer appearance of the continuous capsule is shown. The fatty tissue (yellow, asterisk) covered the anterior part of the ankle and talonavicular joints. Posterior to the insertion of the posterior tibialis tendon (PT), the cartilaginous tissue, normally referred as the SML (aqua area, dagger), located to form the gliding floor of the tendon (white dotted area). Black dotted area indicates the gliding floor of the flexor digitorum longus tendon. **c** The continuous capsule shown in (**b**) is posteriorly reflected to show the inner appearance. The fibrous tissue, normally referred as the deep deltoid ligament (green area), connects between the intercolliculus groove of medial malleolus (black circle) and the depression of the medial talus (section mark). The fatty tissue extended medially between the fibrous and the cartilaginous tissues. The cartilaginous tissue covered the talus head. Red dotted area indicates the capsular attachment on sustentaculum tali (double dagger). *Prox*, proximal; *Dist*, distal

We provided an anatomical description of the medial ankle from the perspective of the joint capsule. In the macroscopic observation, the joint capsule could be detached as a continuous sheet covering the ankle, subtalar, and talonavicular joints. According to the location, there are 3 areas of macroscopically and histologically distinct variations within the continuous capsule. First, the anterior part of the joint capsule consisted of fatty tissue. Second, between the medial malleolus and the talus, the capsule showed a strong connection, similar to fibrous tissue. Third, on the subtalar and talonavicular joints, the capsule covered the joints as cartilaginous tissue. In addition, the outer side of the fibrous and cartilaginous tissues, as described above, formed the sheath floor of the PT tendon (Fig. 7).

The deltoid ligament has been well-studied in the field of medial ankle joint anatomy. In the textbook of Sarrafian’s anatomy (Sarrafian 1993), 14 studies reported different descriptions of the components of the deltoid ligament. However, there remains a paucity of conclusive studies (Boss and Hintermann 2002; Campbell et al. 2014; Panchani et al. 2014). In terms of the variable interpretations of the components of the deltoid ligament, Boss et al. pointed out the difficulty in clearly separating each ligamentous component (Boss and Hintermann 2002). Previous reviews concluded that difficulty in identifying the components may arise from interobserver variability due to artificial divisions of the ligaments (Golano et al. 2010; Savage-Elliott et al. 2013). Thus, the conventional method for detecting ligamentous components might limit our understanding of the ligament morphology.

The current study presents a different perspective based on the joint capsule instead of identifying the ligamentous components. As a result, we could detach the joint capsule as a continuous sheet from the anterior to medial ankle. In the continuous capsule, the fatty tissue at the anterior ankle as described above changed into fibrous tissue as it moved medially to the area between the medial malleolus of the tibia and the talus. Given that the anterior capsule has been described to medially continue to the anterior border of the deep deltoid ligament (Sarrafian 1993), the fibrous part of the continuous capsule could be interpreted as the previously recognized deltoid ligament. Generally, the joint capsule consists of the fibrous and synovial connective tissues, and the thickness and fiber orientation of the capsule depends on the stress it endures (Ralphs and Benjamin 1994). Therefore, the deltoid ligament could be interpreted to be the part of the capsule that is rich in fibrous structures, due to the mechanical stress connecting the medial malleolus and the talus, rather than a specific and consistent “ligamentous” structure. In addition, the synovial and fibrous parts could be interpreted to complement each other to form the continuous capsule. We hypothesized that this might explain why the reported number of bundles and the morphology of the deltoid ligament are variable and still inconclusive in studies that aim to evaluate ligamentous components.

On the other hand, the deltoid ligament has been commonly understood to be composed of two layers: superficial and deep (Milner and Soames 1998). Savage-Elliott et al. (Savage-Elliott et al. 2013) reported that the synovial tissue existed at the border of the deep and superficial deltoid ligament. On histological analysis in the current study, we showed that the synovial tissue filled the gap between the fibrous and cartilaginous tissues at the medial side of the talus. This finding was supported by the description provided by Savage-Elliott et al., which could be the reason why the deltoid ligament could be separated into superficial and deep layers by consensus.

Several previous reports have discussed the attachment location of the deltoid ligament. Boss et al. (Boss and Hintermann 2002) showed the approximate insertions of the components. Campbell et al. (Campbell et al. 2014) demonstrated attachments of the components with a measurement device. Cromeens et al. (Cromeens et al. 2015) reported 11 attachment sites mapped using a microscribe 3D digitizer. Although these studies showed the location of each component, there were limited descriptions of the bony morphology to which the components of the ligament attached. On the basis of the present study, there were unique morphologic characters of the bone corresponding to the fibrous part of the continuous capsule, normally referred to as the deep deltoid ligament, in both the tibia and talus. On the tibial side, the fibrous part attached to the depression between the anterior and posterior colliculus, which was previously referred to as the intercolliculus groove. At the talar side, the fibrous part attached to an area that also appeared to be a depression. As a result of the mechanical force loaded by the fibrous part of the continuous capsule, the tibial intercolliculus groove and talar depression sterically formed. Campbell et al. (Campbell et al. 2014) and Cromeens et al. (Cromeens et al. 2015) similarly reported that the deep posterior tibiotalar ligament attaches to the talus with the area of 129.6 and 140.9 mm^2^, respectively. These previous data approximately support that the dimensional measurement of the medial talar depression in the present study, which was thought to correspond to the talar attachment of the deep posterior tibiotalar ligament, was 8.9 mm width and 17.5 mm length, which was speculated to be approximately the same size.

The spring ligament, which connects between the calcaneus and the navicular bone, has been classically separated into two structures: the larger superomedial ligament (SML) and the smaller inferoplantar ligament. The SML has received attention because of its static support for the head of the talus via maintenance of the longitudinal arch. A previous histological study showed the absence of elastin fibers and cartilaginous appearance of the SML (Vadell and Peratta, 2012). In the current study, the cartilaginous tissue in the continuous capsule formed an articular surface in contact with the head of the talus and connected the calcaneus and the navicular. With regard to location and appearance, the traditional SML corresponds with this cartilaginous tissue in the continuous capsule of the current study.

The proximal connections between the medial malleolus and SML have been referred to as the tibiospring ligament, which represents one component of the superficial deltoid ligament (Boss and Hintermann 2002; Campbell et al. 2014; Milner and Soames 1998; Panchani et al. 2014). On the basis of the results of the current study, the tibiospring ligament corresponds to a part of the continuous capsule that connects the medial malleolus and the cartilaginous tissue. Cromeens et al. (Cromeens et al. 2015) reported that the tibiocalcaneonavicular ligament included the previously described tibiocalcaneal ligament, tibionavicular ligament, tibiospring ligament, and SML. This might support our claims that each individual ligament could be regarded as a part of the continuous capsule.

The SML has been considered to have no attachment to the talus (Vadell and Peratta, 2012). Although the tibiocalcaneonavicular ligament as described by Cromeens et al. (Cromeens et al. 2015) is inclusive of this complex structure, it did not refer to a connection with the talus. We found that fatty tissue deep to the cartilaginous tissue connected the SML to the talus. This fatty tissue filled the gap between the fibrous tissue, which was normally referred to as the deep deltoid ligament, and cartilaginous tissue, which was normally referred to as the SML, macroscopically and histologically (asterisk in Figs. 2a and 6d, e, respectively). The synovial fatty tissue described by Savage-Elliott et al. (Savage-Elliott et al. 2013), which existed at the border between the deep and superficial deltoid ligament, supported the presence of the fatty tissue in the present study. The fatty tissue in the present study seemed to connect the cartilaginous tissue to the talus, which had a fibrous connection only with the medial malleolus. In addition, the fatty tissue is generally rich in nerve endings, such as that of the tarsal sinus (Rein et al. 2015). The fatty tissue deep to the cartilaginous tissue might correlate with pain around the medial malleolus recognized as the initial pain of PTTD.

There are few anatomic studies of the capsule structure around the medial side of the ankle joint. In terms of the anterior capsule, only Tol et al. (Tol and van Dijk 2004) described the attachment of the anterior capsule and reported the histological features to include fatty tissue in the synovial membrane. Similarly, the present study revealed that the anterior part of the continuous capsule consisted of synovial and fatty tissue, which was clearly separate from the fibrous and cartilaginous tissues located posteriorly. In addition, we demonstrated the precise widths and locations of the capsular attachment on the tibia, talus, and navicular bones, which were supported by the previous reports as rarely described.

The results of the current study have two clinical implications. First, the findings of the medial continuous capsule in the current study could be useful in determining the etiology of AAFD. The current study shows that the conventional deltoid and spring ligaments joined with each other to form the sheath floor of the PT tendon as a continuous sheet. The anatomical proximity of these structures could explain the multifactorial progression of AAFD. Second, describing the location and width of the anterior capsule might be helpful for understanding the pathology and imaging of anterior impingement syndrome. Anterior ankle impingement, which is associated with both tibiotalar osteophytes and/or soft tissue impingement, is well known to cause anterior ankle pain and restricted dorsiflexion (Biedert 1991). Traction force of the anterior capsule could be a possible pathomechanism resulting in osteophyte formation (Williams and Brandt, 1984). Furthermore, traumatic sprains with capsular tearing also cause the formation of scar tissue, leading to soft tissue impingement (Ross et al. 2017). Hence, the anterior capsule has been considered to have a crucial association with anterior impingement syndrome. The fact that the anterior capsule consisted of fatty tissue with substantial attachments on the tibia and talus might correlate with the pathology of anterior impingement syndrome.

The limitations of the study are as follows. First, this was a purely anatomical study limited to healthy samples. Thus, we could not draw conclusions concerning the mechanism of AAFD and would need to include biomechanical studies or imaging of clinical cases based on our anatomical findings. Second, all investigated cadavers were from elderly patients, and this age group does not match the peak age of onset of medial ankle injuries. Therefore, the histological features in the present study could be inconsistent in the younger specimens. Third, we could not confirm the history of surgeries and trauma in the medial ankle from the recorded information. Thus, there is a possibility that postoperative or posttraumatic specimens were included, which could not be identified during the dissections.

## Conclusion

The capsules of the ankle, subtalar, and talonavicular joints could be detached as a continuous sheet. The continuous capsule consisted of 3 different tissues: the fatty and synovial tissue, corresponding to the anterior capsule; the fibrous tissue, corresponding the deep deltoid ligament; and the cartilaginous tissue, corresponding to the SML of the spring ligament. The deltoid and spring ligaments could be interpreted to be a part of the joint capsule.
